# Risk of Thrombosis in Women Undergoing In Vitro Fertilization: A Narrative Review

**DOI:** 10.3390/jcm14041053

**Published:** 2025-02-07

**Authors:** Elvira Grandone, Victoria Bitsadze, Jamilya Khizroeva, Elena Chinni, Mario Mastroianno, Luigi Nappi, Maria Tretyakova, Natalia Makatsariya, Kristina Grigoreva, Nilufar Gashimova, Arina Lazarchuk, Daredzhan Kapanadze, Tatyana Polyakova, Anastasia Shatilina, Elizaveta Lyadnova, Armen Blbulyan, Nart Kuneshko, Marina Zainulina, Grigoris Gerotziafas, Alexander Makatsariya

**Affiliations:** 1 Department of Obstetrics, Gynecology and Perinatal Medicine, The I.M. Sechenov First Moscow State Medical University (Sechenov University), Trubetskaya Str. 8-2, 119435 Moscow, Russia; vikabits@mail.ru (V.B.); jamatotu@gmail.com (J.K.); tretyakova777@yandex.ru (M.T.); makatsariya@gmail.com (N.M.); grigkristik96@gmail.com (K.G.); nelya.94@yandex.ru (N.G.); arina.lazarchuk@mail.ru (A.L.); sam.miel@ya.ru (T.P.); shatilina1110@gmail.com (A.S.); elizavetalyadnovaaa@gmail.com (E.L.); grigorios.gerotziafas@inserm.fr (G.G.); gemostasis@mail.ru (A.M.); 2 Thrombosis and Hemostasis Unit, Fondazione IRCCS Casa Sollievo della Sofferenza, 71013 San Giovanni Rotondo, Italy; e.chinni@operapadrepio.it (E.C.); m.mastroianno@operapadrepio.it (M.M.); 3 Department of Medical and Surgical Sciences, Institute of Obstetrics and Gynecology, University of Foggia, 71122 Foggia, Italy; luigi.nappi@unifg.it; 4 Center of Pathology of Pregnancy and Hemostasis Medlabi, 340112 Tbilisi, Georgia; medlabimedlabi@gmail.com; 5 Research Center of Maternal and Child Health Protection, 22 Mashtots Avenue, Yerevan 0002, Armenia; belbulyan@mail.ru; 6 Moscow’s Region Odintsovo Maternity Hospital, 143003 Odintsovo, Russia; drnartfaruk@mail.ru; 7 Snegirev Maternity Hospital No. 6, 5 Mayakovskogo Str., 192014 Saint Petersburg, Russia; zainulina@yandex.ru; 8 Obstetrics and Gynecology Department, Pavlov First Saint Petersburg State Medical University, Health Ministry of Russian Federation, 6/8 Lev Tolstoy Str., 197022 Saint Petersburg, Russia; 9 Saint-Antoine Research Center (CRSA), University Institute of Cancerology (UIC), Sorbonne University, INSERM UMR_S_938, 34 Rue du Crozatier, 75012 Paris, France; 10 Thrombosis Center, Tenon-Saint Antoine University Hospital, Hôpitaux Universitaires Est Parisien, Assitance Publique Hôpitaix de Paris (AP-HP), 4 Rue de la Chine, 75020 Paris, France

**Keywords:** thrombosis risk, ovarian stimulation, In Vitro Fertilization (IVF)

## Abstract

This narrative review summarizes the available literature on the association between In Vitro Fertilization (IVF) treatments and thrombosis, focusing on epidemiology and pathophysiology. Thrombosis is a rare IVF-related complication, with an incidence of approximately 0.2%, dramatically increased by ovarian hyperstimulation syndrome (OHSS). Arterial thrombosis, primarily associated with OHSS, is a rare and early event, while venous thrombosis, although more common, remains a rare complication of IVF. Venous thrombosis often affects the upper body. The thrombotic risk is higher during the first trimester of pregnancy obtained through IVF. This review discusses the impact of risk factors such as OHSS, thrombophilia, obesity, advanced maternal age, and polycystic ovarian syndrome, which predispose women to thromboembolic events during and after IVF stimulation.

## 1. Introduction

In 1978, Robert Edwards and Patrick Steptoe announced the birth of the first baby resulting from in vitro fertilization (IVF). Today, over 8 million babies have been born through IVF [1]. In 2019, the European IVF-monitoring Consortium (EIM) for the European Society of Human Reproduction and Embryology (ESHRE) reported 1487 clinics offering assisted reproductive technologies (ARTs) in 40 European countries, with a total of 1,077,813 treatment cycles, including 160,782 IVF cycles [2].

Ovarian stimulation is a critical step in IVF treatment, enabling precise timing of insemination and the retrieval of multiple oocytes [3]. Controlled ovarian stimulation (COS) for IVF involves administering exogenous gonadotropins, including follicle-stimulating hormone (FSH) and luteinizing hormone (LH), often in combination with gonadotropin-releasing hormone (GnRH) agonists or antagonists. Ovarian maturation is then triggered using human chorionic gonadotropin (hCG) [3]. However, excessive ovarian response to these exogenous gonadotropins has been associated with an increased risk of thrombotic events [4,5].

Despite these concerns, the available evidence on this issue remains limited and primarily derived from small studies, case series, and individual case reports. Systematic reviews and meta-analyses conducted to date have mostly included observational studies, such as small-cohort and case–control studies, which limits the strength of their conclusions [6,7].

This review highlights both areas of consensus and ongoing controversy in the field. Crucially, it underscores the significant gaps in the existing literature and the urgent need for large-scale, high-quality prospective studies to better understand the relationship between ovarian stimulation and thrombotic risk. Future research should aim to provide more robust evidence to guide clinical practice and improve patient safety during IVF treatment.

### Methodology

This narrative review examines the association between ART and thrombotic complications. We explored relevant studies published in peer-reviewed journals, focusing on the epidemiology, pathophysiology, and clinical outcomes of thrombotic events in women undergoing IVF. Emphasis was placed on ovarian hyperstimulation syndrome (OHSS), a key complication linked to both arterial and venous thrombotic risk.

Data were extracted from cohort studies, case reports, and reviews, comparing thrombotic event rates in IVF-treated populations to those in the general population. Anatomical localization and timing of thrombotic events were analyzed to identify patterns and risk factors, with a focus on the roles of hCG, hormonal changes, and fluid dynamics.

## 2. Thrombosis in IVF

ART has become an integral part of routine care in many countries. However, its widespread adoption has led to a significant increase in risk factors for pregnancy-related thrombosis. This rise is largely attributed to the active introduction of new medical technologies that were either unavailable 30 years ago or whose potential role in thrombosis development had not been thoroughly studied.

### 2.1. OHSS and Thrombotic Risk

**Figure 1:**
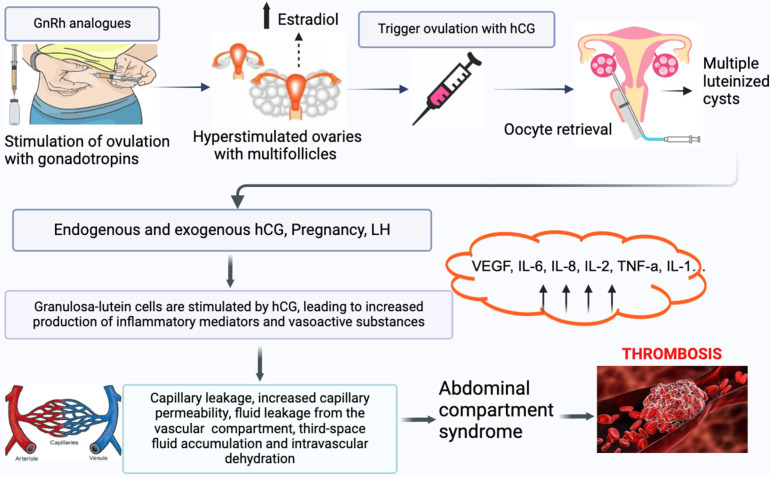
Pathophysiology of OHSS. GnRH—gonadotropin-releasing hormone; hCG—human chorionic gonadotropin; LH—luteinized hormone; VEGF—vascular endothelial growth factor; IL-6—interleukin-6; IL-8—interleukin-8; IL-2—interleukin-2; TNF-a—tumor necrosis factor-alpha; IL-1—interleukin-1.

Severe IVF-related complications are rare [8]. However, the increase in IVF treatments has led to more women experiencing complications. The most common and serious complication is OHSS [9], characterized by ovarian enlargement, increased vascular permeability, and intravascular dehydration with fluid accumulation in the third space (Figure 1). Moderate or severe OHSS occurs in 3–8% of successful IVF cycles and significantly increases the risk of both arterial and venous thrombosis [5]. The incidence of venous thrombosis in IVF cycles is reported as 0.1–0.5% [10,11,12], while arterial thrombosis is even lower [13,14]. OHSS increases thrombotic risk by about 2% in absolute terms [13,15]. It remains a significant complication inART, necessitating effective strategies to mitigate its risk. There are several promising interventions for reducing OHSS risk, including the individualized administration of follitropin delta, which has demonstrated efficacy in lowering moderate-to-severe OHSS risk without compromising reproductive outcomes. Despite the potential benefits of some strategies, such as adjusted gonadotropin dosing and low-dose hCG administration, the quality of supporting evidence often remains suboptimal [16].

### 2.2. Arterial Thrombosis in IVF Cycles

Arterial thrombotic events (ATEs) are rare IVF-associated complications [17,18]. On average, ATEs occur 10 days after the last hCG treatment [19,20,21] (Table 1). Cohort studies in Denmark, France, and Italy have explored the association between ATEs and IVF, showing ATEs in one to two ovarian stimulation cycles [11,15,22]. Notably, multiple thromboses can occur in the same cycle; for instance, simultaneous arterial radial and mesenteric vein thromboses were reported in one woman with mild OHSS during her fifth cycle [11]. Several case reports have linked ATEs with OHSS [19,21], and Chan WS reported that 95% of ATEs occurred with OHSS [21]. However, the relationship between ATEs and OHSS has not been explored well. A French cohort study of 2490 OHSS cases found a 3% prevalence of ATEs (n = 78) [20], while a Danish cohort study of 30,884 women undergoing 75,141 treatments found no correlation between OHSS and ATE [14]. The authors reported a 6-month incidence rate of 0.8, which is not significantly different from that observed in a reference population of young women [reference: 2.5; IRR = 0.36, 95% CI: 0.04–1.30]. Women with severe OHSS were treated with low-molecular-weight heparins (LMWHs) to reduce VTE risk [14]. Prophylaxis could have impacted both VTE and ATE risks.

### 2.3. Venous Thrombosis in IVF Cycles

Venous thromboembolism (VTE), defined as deep vein thrombosis with or without pulmonary embolism (PE), is more frequently reported than arterial thrombosis but remains a rare IVF-related complication. The estimated prevalence is 0.5%, corresponding to 1.6 per 100,000 cycles/woman [10]. VTE often occurs 26.6 days after hCG administration for ovulation induction cycles (Table 1) [18]. A prospective French cohort study showed a higher risk of VTE in both unsuccessful and successful fertility treatment cycles [20]. VTE was observed in 75 out of 705,186 unsuccessful cycles and 207 out of 82,821 successful cycles. Conventional ovarian stimulation, but not clomiphene citrate-induced ovarian induction, was associated with an age-adjusted Incidence Rate Ratios (IRRs) of 1.74, 95% CI: 1.30–2.34 [20]. However, discrepancies exist regarding the significant VTE risk associated with IVF cycles [14]. An Italian cohort study reported VTE events in 2/1518 (1.3‰) unsuccessful cycles compared to 3/318 (9.4‰) successful cycles (two-tailed Fisher exact test, *p* = 0.04, OR 0.14, 95% CI: 0.02–1.02) [22]. Conversely, Hansen et al. found no increased VTE risk in 30,884 Danish women within 6 and 12 months after ART (IRR: 0.95, 95% CI: 0.38–1.95 and IRR: 1.27, 95% CI: 0.69–2.12, respectively) [14].

### 2.4. Anatomical Localization

Many studies report that ischemic stroke is the most frequent arterial thrombotic event [19] (Table 1), with an unfavorable prognosis, especially in women developing stroke following OHSS-induced hormonal treatment [24,25,26,27]. VTE has been reported at unusual sites (Table 2), such as the upper limbs, neck [15,18,28,29,30], and cerebral veins [23,31,32,33]. Isolated PE is more frequently associated with IVF than other conditions [11,20,22,23,34,35,36]. The reason for the prevalence of thrombosis in unusual sites is not known. It has been hypothesized that hemostatic changes during controlled ovarian stimulation increase peritoneal fluid, drained through the thoracic duct into the subclavian veins [37], leading to a local environment with an increased VTE risk in the upper body [38]. hCG, previously used for final oocyte maturation and ovulation, contributes to hemostatic modifications by decreasing anticoagulants like protein C, protein S, and antithrombin and increasing endothelial markers of vascular damage such as thrombomodulin [39].

Branchial cysts near the jugular or subclavian veins may also increase VTE risk, particularly during OHSS when these cysts are fluid-filled and impair blood circulation [30].

### 2.5. Thrombophilia

Case reports and series suggest that IVF, especially with OHSS, creates a hypercoagulable state, triggering thrombosis [18,34]. Individual VTE risk can increase with inherited or acquired thrombophilia, as shown by Dulitzky et al., who reported an 85% prevalence of thrombophilia in women with severe OHSS, indicating a role for thrombophilia in VTE post-OHSS [23]. However, a prospective observational study did not confirm severe OHSS cases among carriers of common inherited thrombophilia (factor V Leiden and prothrombin gene G20210A mutation) [42]. Similarly, Grandone et al. did not find a role for inherited thrombophilia in IVF-associated thrombosis [10,34,40] in low-risk women. An observational study of 305 women undergoing ovarian stimulation found that increased homocysteine levels—but not other thrombophilias—were significantly associated with VTE (OR: 15.2, 95% CI: 2.0–115.0) [10]. Data from the RIETE registry comparing IVF-related VTEs to other VTEs in childbearing-aged women also did not show an increased VTE risk in those with thrombophilia [34].

### 2.6. Other Maternal Risk Factors

Maternal age is a significant factor influencing the risk of ATE and VTE. Women over 35 years of age have a substantially higher risk of both ATE and VTE during in vitro fertilization (IVF) procedures [10,12]. A large population-based study reported a two-fold increase in VTE risk among women aged 40 years (OR: 2.1; 95% CI: 1.3–3.7) compared to younger women [12]. However, a Danish cohort study did not corroborate these findings [40]. Hansen et al. found that maternal age did not significantly impact VTE risk during pregnancy, although the postpartum period posed the highest VTE risk for women over 35 years [Relative Risk (RR): 2.4; 95% Confidence Interval (CI): 0.8–7.7)] [40]. Additionally, a body mass index (BMI) greater than 30 kg/m^2^ further increases individual VTE risk, with odds ratios (ORs) ranging from 1.23 (95% CI: 1.01–1.49) to 3.2 (95% CI: 2.2–4.6) [10,12]. It is hypothesized that elevated estrogen levels in obese women may enhance coagulation factors, thereby increasing the risk of thrombosis [11]. Currently, there are limited systematic data on the impact of smoking on ATE and VTE risk during or after IVF treatments.

Women with polycystic ovarian syndrome (PCOS) are particularly vulnerable to thrombotic complications during IVF pregnancies [40,43]. PCOS is linked to increased thrombotic risk due to underlying metabolic and hormonal abnormalities, including hyperestrogenism, insulin resistance, and chronic low-grade inflammation, all of which contribute to a prothrombotic state. Moreover, the higher incidence of OHSS in women with PCOS—characterized by increased vascular permeability and hemoconcentration—further amplifies the risk of thrombotic events, particularly in the context of ART [44]. A systematic review and meta-analysis of observational studies revealed that women with PCOS have a five-fold higher risk of VTE compared to those without PCOS. The analysis also provided detailed risk estimates for other contributing factors [7] (Table 3).

### 2.7. Risk of VTE in Pregnancy After IVF

**Figure 2:**
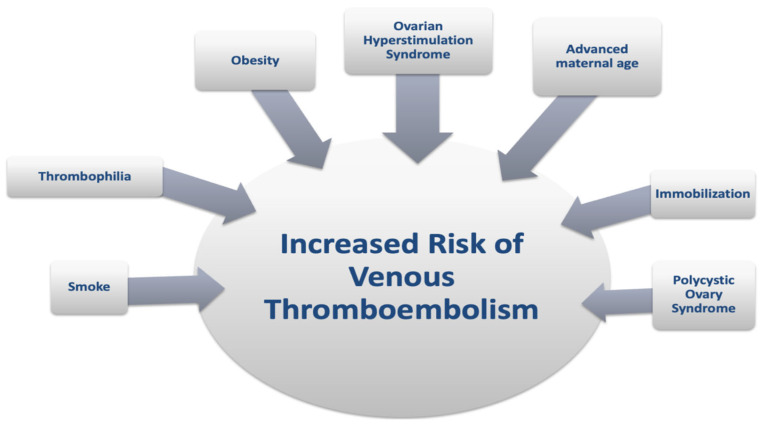
Risk factors predisposing to thromboembolic events during in vitro fertilization.

Robust data indicate that VTE risk in pregnancies following IVF is significantly higher than in natural conceptions [11,40,41]. Many risk factors predispose women to thromboembolic events during pregnancies following successful IVF (Figure 2). Thrombophilia, obesity, smoking, advanced maternal age, and immobilization are known VTE pregnancy-associated risk factors [28,45]. IVF procedures increase this risk three- to four-fold compared to natural conception [40,41,43]. OHSS remains the main factor associated with VTE, with a 100-fold increased risk in pregnancies following successful stimulation [11,12,18,40,41]. The risk persists from one week post-embryo transfer until the end of the first trimester and can last several weeks after OHSS resolution [18,46]. Thus, guidelines recommend LMWH prophylaxis for three months post-OHSS resolution in women with severe OHSS [47,48,49] (Table 4).

Estradiol elevation during IVF has been linked to the downregulation of fibrinolysis, increasing the risk of thrombosis [51,52]. Higher estradiol levels are associated with elevated factor VIII and von Willebrand factor antigen and activity, as well as reduced ADAMTS13 antigen and activity, all of which further contribute to an elevated thrombotic risk [53].

In contrast, pregnancies achieved through frozen–thawed embryo transfer—where ovulation induction and estrogen elevation are not involved—appear to have a VTE risk comparable to natural conception. A Swedish study found no increase in VTE risk associated with frozen–thawed embryo transfers [12]. Additionally, a recent cohort study reported that frozen–thawed embryo transfer significantly reduces first-trimester VTE risk [35]. The study observed that the incidence of VTE after fresh embryo transfer was more than eight times higher than in natural conception, while no increase was noted with frozen–thawed embryo transfer [35]. These findings suggest that frozen–thawed embryo transfer may help lower maternal VTE risk following IVF.

### 2.8. Strengths and Limitations

A notable strength of this review is the comprehensive exploration of different thrombotic risks, particularly the detailed focus on OHSS as a key factor influencing both arterial and venous events. This review draws on a variety of cohort studies, case reports, and observational studies, which offer valuable insights into the incidence, timing, and anatomical localization of thrombotic events.

The limitations of the available studies must also be considered. Many of the studies are observational or retrospective, limiting the ability to draw definitive causal conclusions. Moreover, discrepancies between studies regarding the association between OHSS and arterial thrombosis, as well as inconsistent findings on the role of various risk factors, highlight the need for further research.

This review also points out the lack of systematic data on smoking and its potential impact on thrombotic risk, as well as the absence of robust data on the effectiveness of preventive strategies.

## 3. Conclusions

Thromboembolism remains a rare but significant complication in IVF cycles, with VTE being more common than arterial thrombotic events. OHSS emerges as the primary risk factor for thromboembolism, with both arterial and venous events more likely to occur in women experiencing this complication. As such, current guidelines emphasize thromboprophylaxis, particularly for those with severe OHSS. The available data on the role of inherited and acquired thrombophilia in thromboembolic risk remain inconclusive, especially in women with multiple risk factors.

Further research is necessary to clarify the mechanisms linking thrombophilia to IVF-related thromboembolism, including the effects of hormonal treatments and other pre-existing conditions. Additionally, while emerging evidence suggests that frozen–thawed embryo transfer may lower the thromboembolic risk compared to fresh embryo transfer, more rigorous studies are needed to confirm these findings. Future clinical recommendations should focus on personalized thrombosis prevention strategies, considering individual patient risk factors such as age, BMI, and the presence of conditions like PCOS, to optimize maternal outcomes during IVF treatments.

## Figures and Tables

**Table 1:** Available data on incidence, anatomical localization, and timing of arterial thrombotic events.

	Ref.	Author	Study Design	IVF Population (n)	Cycles (n)	ATEs (n)	Incidence of ATEs per Cycle (n)	ATE Localization	Timing of ATEs (Mean Days after hCG)
2002	[23]	Dulitzky, M.	Cohort	61	na	1	na	Ischemic stroke	na
2004	[10]	Grandone, E.	Case–control	305	747	3	0.40%	Ischemic stroke, upper extremity	na
2007	[17]	Girolami, A.	Review case series	34	na	34	na	Ischemic stroke, carotid, AMI, peripheral, other	13.7
2009	[18]	Chan, W.S.	Review case series	96	na	35	na	Ischemic stroke, neck, AMI, peripheral	10.7
2012	[14]	Hansen, A.T.	Cohort	30,884	75,141	2	0.003%	na	na
2017	[19]	Yang, S.	Case series	38	na	29	na	Ischemic stroke	8
2019	[20]	Filipovic- Pierrucci, A.	Cohort	277,913	788,007	78	0.01%	na	na

IVF: in vitro fertilization. ATE: arterial thrombosis. hCG: human gonadotrophin; AMI: acute myocardial infarction; na: not available.

**Table 2:** Available data on incidence, anatomical localization, and timing of venous events.

	Ref.	Author	Study Design	IVF Population (n)	Cycles (n)	VTEs (n)	Incidence of VTEs per Cycle (n)	VTE Localization	Timing of VTEs (Mean Days After hCG)
1993	[33]	Delvigne, A.	Case–control	384	na	1	0.01%	CSVT	na
1995	[32]	Kodama, H.	Case series	23	1316	1	0.08%	CSVT	11
1998	[36]	Abramov, Y.	Case–control	163	163	4	2.50%	PE	na
1998	[15]	Serour, G.I.	Case–control	2924	3500	4	0.12%	upper extremity	na
1998	[31]	Aboulghar, M.A.	Case reports	2	2	2	100%	CSVT	5
2002	[23]	Dulitzky, M.	Cohort	61	na	2	na	PE	na
2004	[10]	Grandone, E.	Case–control	305	747	2	0.30%	CSVT; SVT	na
2006	[28]	Chan, W.S.	Review case series	37	2500	37	1.48%	upper extremity; neck	28
2009	[30]	Salomon, O.	Case series	5	na	5	na	upper extremity; neck	16
2009	[18]	Chan, W.S.	Review case series	96	na	61	na	upper extremity; neck	26.6
2012	[12]	Rova, K.	Cohort	19,194	na	32	na	na	45
2012	[14]	Hansen, A.T.	Cohort	30,884	75,141	7	0.009%	na	na
2012	[29]	Fleming, T.	Case series	2	2	2	100%	neck	39.5
2013	[11]	Henriksson, P.	Cohort	23,498	na	99	na	PE; other not specified	na
2014	[40]	Hansen, A.T.	Cohort	18,787	na	36	na	PE; other not specified	na
2015	[41]	Villani, M.	Cohort	234	684	6	0.88%	PE; lower limb	na
2017	[19]	Yang, S.	Case series	38	na	2	na	CSVT	8.33
2018	[22]	Villani, M.	Cohort	661	1836	5	0.27%	PE; lower limb	na
2018	[34]	Grandone, E.	Cohort	41	ns	41	na	PE; lower limb, upper extremity	na
2019	[20]	Filipovic-Pierrucci, A.	Cohort	277,913	788,007	282	0.04%	PE; lower limb	na
2020	[35]	Olausson, N.	Cohort	30,328	na	161	na	Lower limb PE, SVT, CSVT, others	na

IVF: in vitro fertilization. VTE: venous thromboembolism. hCG: human gonadotrophin; CSVT: cerebral sinus vein thrombosis; PE: pulmonary embolism; SVT: splanchnic vein thrombosis; na: not available.

**Table 3:** Magnitude of VTE risk according to various risk factors ^.

Risk Factor	Magnitude of the Risk
OHSS	RR: 14.83; 95% CI: 0.86–255.62
IVF	OR 1.77, 95% CI: 1.41–2.23 HR 4.99, 95% CI: 1.24–20.05
Hyperhomocysteinemia	OR: 15.2; 95% CI: 2.0–115.0
PCOS	RR: 4.8; 95% CI: 1.7–13.4
Successful ART leading to pregnancy	OR: 13.94; 95% CI: 1.41–137.45

^ According to ref. [7]. VTE: venous thromboembolism; OHSS: ovarian hyperstimulation syndrome; RR: Relative Risk; CI: Confidence Interval; IVF: in vitro fertilization; OR: odds ratio; HR: hazard ratio; PCOS: polycystic ovarian syndrome; ART: assisted reproductive technology.

**Table 4:** Drugs and precautions to prevent VTE.

Guideline	Intervention	Grade of Evidence
ACCP 2012 [47]	LMWHs	2C
RCOG 2015 [49]	LMWHs	2+/C
British Fertil. Society 2024 [50]	Subcutaneous LMWHs are the anticoagulant of choice	A
British Fertil. Society 2024 [50]	If risk factor(s) for VTE, transfer single embryo	D

VTE: venous thromboembolism; LMWHs: low-molecular-weight heparins.
